# Becoming a Psychotherapist: Learning Practices and Identity Construction Across Communities of Practice

**DOI:** 10.3389/fpsyg.2021.770749

**Published:** 2022-01-14

**Authors:** Francesca Alby, Cristina Zucchermaglio, Marilena Fatigante

**Affiliations:** Department of Social and Developmental Psychology, Sapienza University of Rome, Rome, Italy

**Keywords:** groups, community of practice, situated learning, group psychotherapists, Italy, professional identity

## Abstract

Within a perspective that views groups as communities of practice and sites of construction of knowledge, learning, and identity, this article aims to explore the contribution that participation in different groups over the course of one’s life provides to the development of the professional practices of psychotherapist trainees enrolled in the C.O.I.R.A.G. school, an Italian graduate program in group psychotherapy. Through qualitative analyses of 10 semi-structured interviews, our study empirically shows that by participating in groups, the trainees not only learn the practices of that group but also develop a sort of meta-learning which takes place across groups. The results highlight that: (1) Transversality, duration, and informality are found to be the group properties with the highest formative value; and (2) Learning practices across different groups have common characteristics: are organized around complex topics of group life (e.g., how to manage conflicts, how to join and leave groups, etc.), began in early group experiences, are in continuous evolution, are associated with a critical event, and a negative affect. At the same time, it seems that these critical events are exactly what triggered and sustained the learning practices. Data from the interviews also showed how professional identities are constructed as the outcome of learning in different communities of practice. The study outlines how the experience made in different groups is elaborated in and through meaningful self-narratives, highlighting them as a fundamentally collective and culturally shaped sense-making process. Overall, these results contribute to a better understanding of learning processes as situated and jointly constructed through multiple group participations over time. Furthermore, they contribute to highlighting the role of self-narratives as a primary way through which trainees shape their identity as self-reflexive professionals who are competent in reading group dynamics. Directions for future research and suggestions for psychotherapist training paths are outlined in the conclusions.

## Introduction

What do we learn in the groups we participated in throughout our lives? How do these experiences affect the acquisition of useful skills for the profession of group psychotherapist? We contribute to answering these questions with a qualitative exploratory study that we frame within the literature on situated learning (Resnick, 1987; Lave, 1988; Lave and Wenger, 1991; Rogoff, 1995). According to this research tradition, learning and knowing, as well as social identity, are the result of participation in communities of practice. As Wenger has suggested, participation refers to “not just to local events of engagement in certain activities with certain people, but to a more encompassing process of being active participants in the *practices* of social communities and constructing *identities* in relation to these communities” (Wenger, 1998, p. 4; emphasis in original).

In his analysis of an insurance company, Wenger (2000) described how a complex dynamic between participation and non-participation underlies the work practices and the professional identity of the employee community. In this framework, participation (and non-participation) is not just a physical action or event; it involves both action (“taking part”) as well as connection with practices and with others (Wenger, 1998). Since 1991, when it first appeared (Lave and Wenger, 1991) and over the years, the concept of community of practice has been a powerful tool for understanding the social and learning processes, and the perpetuation of a practice in time. This concept helps us to conceive groups not as social objects with defined boundaries, roles, rules, but as forms of joint practicing (Orr, 1996; Wenger, 1998; Gherardi, 2009). The practice, from this view, is what shapes the group, what holds together a configuration of people, artifacts, and social relations. Practices are not, therefore, just mere descriptions of what people do; they are knowing, meaning-making, identity-forming, and order-producing activities (Chaiklin and Lave, 1993; Schatzki, 2001; Gherardi, 2006; Nicolini, 2009). Many studies describe the interplay between learning practices and the construction of social and professional identities in communities of practice (Alby and Zucchermaglio, 2006, 2008, 2009; Zucchermaglio and Alby, 2012, 2014, 2016; Alby et al., 2015; Little, 2015; Gherardi and Rodeschini, 2016; Nicolini et al., 2018). Other studies particularly outline the role played by reflective learning practices in shaping the professional identities (Alby, 2018; Gherardi et al., 2018; Scaratti et al., 2019).

Moreover, the concept of “practice” emphasizes that the process of learning is, at once, social and cognitive, and is linked to participation in groups (Brown and Duguid, 1991, 2001; Little and Horn, 2007). Whereas, in the traditional Cartesian tradition, knowing starts with a subject facing an object, in this perspective the practice itself is seen as the “site of knowing” (Nicolini, 2011). The knower and what is known—the knowing subject and the knowing object—emerge together in practice. The term “site” also indicates that human phenomena are situated within a historical-cultural context that gives them meaning and provides a background understanding of what is relevant, and counts as an object of knowledge (Lave, 1996).

Close to this tradition, Dreier (1999) uses participation as a key concept of his psychological theory in which people are defined as participants in structures of an ongoing social practice. In Dreier’s thought, a context of social practice is understandable, only considering its interrelationships with other contexts. People constantly move from one context to another, continually rearranging their forms of participation in different practices. By moving across contexts, people change their structures of personal relevance, copying with contradictions and building a personal life-trajectory. Contradictions play an important role in personal development; this is often overlooked by most personality theories which focused more on coherent structures of goals, needs, and life plans (Dreier, 1999). By composing a personal life-trajectory across contexts of social practice, individuals face complex and difficult tasks. Contradictions between heterogenous practices and contexts are not easily resolved in a personal synthesis. Developing a personal stance requires an individual’s continual negotiation of “self” within and across multiple communities of practice, which may generate intra-personal tensions, as well as instabilities and changes, within the communities. Within this literature, which overlaps with the work of the actor-network tradition (Latour, 2005; Lindberg and Czarniawska, 2006), individuals are, in fact, depicted as carriers of practices and as a medium through which communities innovate and change.

Drawing on these previous research findings and concepts, this article aims to explore learning practices in and across communities of practice by taking the particular perspective of psychoanalytic trainees, i.e., psychologists enrolled in an Italian graduate school in group psychotherapy. Specifically, our research studies their experiences of participation in and across the groups that they encountered during their life, and the formative value of such experiences for both their social life and future profession.

## Materials and Methods

The data corpus of the study is composed of 10 interviews with students of the C.O.I.R.A.G. (Confederation of Italian Organizations for Analytical Research on Groups) graduate school, a program aimed at training group psychotherapists. The study occurs within a research collaboration agreement between the school of psychotherapy and the university department of the authors. The graduate school is based in Italy and the recruited students are from the section based in Rome. The theorical framework reference of the school is psychoanalysis, and, in particular, its applications to groups (i.e., group analysis, analytic psychodrama, and institutional analysis). In the theoretical perspectives adopted by the school, discursive and intersubjective practices are highly valued and are considered a primary tool for therapeutic works (Lemoine and Lemoine, 1972; Lo Verso and Di Blasi, 2011; Kaës et al., 2012). As the director of the school explained to us in an interview, the school aims to teach the following core professional competencies: ability to think on various levels and see issues from multiple points of view; being open-minded, able to mentally engage with others; and ability to pay attention to collective phenomena and read group dynamics in any context. Moreover, the school requires a personal analysis as part of the training plan.

The program lasts for four years. Educational activities take place for 500 h a year, during the weekends, every two weeks. In addition to lessons on theories and clinical topics, the school uses teaching methodologies based on group devices, in which, unlike lectures, dialog among students, active participation, and autonomous development of concepts and competence is first promoted, using an experiential learning approach. This choice, as we read in the school educational program, was made because the school recognizes that the group is a pivotal tool for developing a shared understanding, deep knowledge, and learning processes. Students therefore participate each year in the numerous meetings of a tutoring group, an experiential group, a clinical observation group, a supervision group, and an annual national residential workshop. All groups are managed by one or more professors.

The interview was inspired by the literature on situated learning and aimed at grasping forms of knowing and learning in groups and across groups in everyday life. The interview, lasting about an hour, was conducted through Zoom by the first author, who presented herself as a researcher in social psychology (indicated as R. in the interview transcripts). The interviews were conducted in Italian and were videorecorded. Interviewees were recruited on a voluntary basis among the students of the last two years of the program.^1^ The participants were informed about the aims of the study and signed a consent form. Interviewees were 6 men and 4 women. Their average age was 34. All of them have a university degree in psychology; they work as private psychologists, cultural educational assistants (AECs) in schools, workers in communities for minors or in the therapeutic communities, and tutors for learning disabilities. The interview guide (see Appendix) explored the experiences of the students in terms of participation in groups of different areas (work, education, and free time/private life). The interview starts by illustrating the purpose of the research (“The research aims to explore how the experiences of participation in groups affect the construction of the professional skills of a psychotherapist”). After reminding the participants to keep in mind the aim of the research, the first instruction was “list the most significant groups that populate or have populated your life in recent years, considering both the working and the educational area, together with the private life and free time.” From this list, the interviewee is then invited to choose the three most relevant groups, one for each area (working life, training, free time, and private life). The following questions were focused on the aim, members, roles, main activities, positive and negative episodes, and learned practices of the group. Question number 12 asked about the practices learned in one group and used in another group to explore connections and paths across groups. We also collected an interview with the school director and documentation from the school website that we used for a background field analysis. The analytical procedures used on the data *corpus* included the following: (1) verbatim transcription of all the interviews, (2) frequency analysis of the elicited groups, and (3) thematic analysis of the interview transcripts (Hsieh and Shannon, 2005; Braun and Clarke, 2006; Alby and Fatigante, 2014) to find learning practices across the groups. We used an inductive process for the construction of coding categories. In the first phase, the researchers read the transcripts of the interviews identifying the categories to be used for coding. In the second phase, these categories were compared and revisited. These codes were subsequently used to carry out the final coding of the transcripts. We coded any reference to learned practices in other groups, which were found to be relevant in the group at hand, for instance “I associate this *(episode about an educational group)* to what I have said so far about the group of friends.” The saturation level of the data was a matter of discussion and the data collected were considered sufficient for qualitative exploratory analysis. The verbatim quotations of the selected interviews in the article are representative of the identified categories. Ethical approval for this study was obtained from the Department Ethics Board (approval number 1332) of the authors.

## Results

We present our findings in two sections: the first one focuses on the characteristics of groups as learning sites for professional practices; whereas the second one focuses on the reflexive narratives and meta-learnings of the interviewees across groups (how and what they learned from participating in various groups throughout their lives).

### Groups as Learning Sites for Professional Practices

This section analyzes the answers to the first two questions, in which the interviewees had to indicate the most relevant groups in their lives for the acquisition of useful practices for the future profession.

In the sphere of private life, interviewees mainly quoted the family groups and groups of friends, which are characterized by longer-term participations than the groups in the other two areas. In the educational area, the class of the graduate school in psychotherapy has been mostly mentioned, while the teaching group devices set up by the school (e.g., experiential group, supervision group, observation groups, and national workshop) are much less mentioned. In the work area, a variety of contexts have been mentioned, all pertinent to the psychological profession (cf. Table 1).

**TABLE 1:** Groups in the areas of free time/private life, education, and work.

Area*	Groups/N	Average members per group	Average duration in years
Free time and private life	- Friends 4 - Family 3 - Theatre 1 - Soccer 1 - Buddhist practice 1	10	17
Education	- Class 6 - Clinical observation (internship) 3 - School supervision group 1	11	2.7
Work	- Community for adolescents 2 - Speech training class 1 - Alcoholics’ group 1 - Community for psychotics 2 - Private supervision group 1 - Psychologists’ group in hospital 1 - AEC (cultural education assistant) group in school 2	16	2.2

*In the groups of the private life and education, the interviewees are involved as ordinary participants, while in the work area, they are involved as psychologists.

Overall, interviewees have selected groups that exhibit some common formative characteristics:

- *Long-lasting and with a constant attendance*. For example, the annual national workshop organized by the school is mentioned but not chosen in the shorter list of the most significant groups because the contact between the participants is not continuous, as reported by an interviewee. The class, which received many mentions, has, in some cases, gone beyond the limit of its formal duration: “I already graduated from the school, but I did not leave the class for real, in the sense that the group has somewhat changed form, it is not anymore a training group within the school but it is a group that has been maintained and it is still there; so in the eyes of the school I got out but really I did not”;
- *Crosses several areas*. This is the case of the graduate school class, which is very much chosen as a significant group in the educational area, but whose transversal nature is often underlined by the interviewees: “as regards the educational area, I choose my class group, even if it is halfway between a training group and a friends’ group, (…) let’s say it is certainly formative, but I honestly also find in it a lot of affection, of friendship that goes beyond the training (…) with two people of this group, and with one in particular, a very strong friendship has formed, that is with a girl I met at the open day, three years have gone by now, and I consider her as if she were practically my sister”; another example is provided by a theater class: “I see theater as something between my training and my social life (…) I feel like all the people I met there, it might be trivial but, bonds are created, knowledge and ties that I have brought with me, even with some who no longer do theater, I still have them in my life anyway, they are still friends with whom I exchange gossip, opinions, evenings”;
- *With informal generative moments*, which create bonds and support learning. An interviewee talks about this, speaking about the school class: “we have always been a group that shared a lot at the lunch breaks which were really a ritual (…) I think we don’t have an official photo with everyone in line, we only have photos eating around the table (…) these were precisely the moments in which we exchanged a lot, let’s say it was all informal but the group was always the one of the class.” Another example is provided by a work group in a hospital: “there were some moments of meeting with the manager, however, in addition to the formal moments, there were also a series of moments that I would say informal but which were not completely informal in the end, we still talked about work and what was happening.”

In moving from one group to another, people bring the practices with them, ways of doing and reasoning about things. On the one hand, this changes the communities, while on the other it creates the opportunity to shape personal learning paths across groups, as discussed in the following paragraph.

### Reflexive Narratives and Meta-Learning Across Groups

Almost all the interviews contain reflexive narratives that connect the experiences made in the different groups (work/education/private life). There were very few cases in which this did not happen, and this is probably due to the contemporaneity of the group experiences; the lack of a temporal perspective makes it difficult to make a comparison.

In such passages from one group to another, the interviewees seem to rely on the practices used in previous groups to manage problems and complex matters of social life. However, the learned practices are not the same; the interviewees reconfigure and refine them through participation in different communities.

The narratives concern central themes of both the group life and the work of a group psychotherapist, such as: how to manage conflicts, how to join and leave groups, the emotions solicited by being in a group, talking and communicating with others, being and behaving as a team, feeling judged by others, and the sense of responsibility. Each topic is the pivot around which the narrative of the interviewee revolves, and it is covered only in that interview.

The properties of such narratives are:

a) the organization around complex issues related to one’s own behavior in groups, issues that have a personal relevance (they are in fact different for each interviewee);
b) the presence of a foundational group, which acts as a point of reference for the experiences in the following groups. This group is often the one that is first mentioned in the interview, but also the temporally oldest (e.g., the family of origin);
c) the rise in a difficult moment, a critical event, to which a negative affect is associated (e.g., “negative feeling,” “discomfort,” and “fear”), and to which a formative and evolutionary value is acknowledged; and
d) the absence of a resolution or a point of arrival; it is rather a progressive work, of continuous regulation and integration, in which different aspects are gradually brought together.

In what follows, we list interview samples that show the highlighted properties of the learning paths across groups. Within the quite rich data, the analytic comments focus on pointing out movements and connections from one group to another.

### T. and the Group Problem-Solving Processes

Interviewee T., 30 years old, chooses to speak, for the sphere of free time, of a group of friends from his hometown, a group that lasted about seven years “which actually broke up just during this last year.” Interviewee T. describes it as his first group of friends and the most important one: “In adolescence I didn’t have many friends (…) I had seen this a bit like a relational wound, let’s say that over time this group has helped me to overcome this thing.”

Initially, it is a group of seven friends, which was then reduced to five because a couple decided to leave the group following an argument. This quarrel is told by T., as a significant event that they made it a recurrence with a name created by mixing the words quarrel and anniversary:

T: We argued with this couple and… since then the five of us who stayed have been very close. So much so that I remember that, after a few months, one or two months after this quarrel, we made ourselves a bracelet, those fortunate ones, all colored, the same for all, all five, we also took a souvenir photo with all hands close together, and… at the beginning let’s say we were very much against the couple who had left, so much that there is one thing that we did, I do not know if it makes you laugh or not though, we, every year, celebrated what we called it the “quarrel-versary” (*litigiversario, in Italian*) (laughs), that is, every year we met again in the same places where we had met that evening with that couple with whom we discussed, remembering the quarrel that had actually allowed us to get closer as a group.
R: What was the quarrel about?
T: In fact, we have never discovered the real motivation. There was a night when-, there were grudges within the group, we knew it. And then one evening, this couple, in particular him, maybe a bit for the alcohol I don’t know, however, he had begun to behave a little, aggressive let’s say, against everyone, saying he was angry with us but we couldn’t understand why. The fact was that, at a certain point, he ran away, on foot, leaving his girlfriend there with us. We then had to take his girlfriend back at home, and then, however, in the following days we asked them several times to meet again in order to clarify, to understand what had happened that evening but they never wanted.

According to T., the quarrel was a difficult but formative moment prompting the group to decide to talk to each other in a more direct and frank way: “from that moment we decided that whatever was wrong, any bitterness between a person and another should be made explicit within the group.” When asked by the interviewer about what he had learned by participating in this group, T. replies: “resolving conflicts or problems in a group context.”

The issue of the quarrel is also recalled by T. later in the interview regarding the graduate school class, which he chose as the most important group in the training field. The topic of joining and leaving the group and the difficulties they entail were also reissued. Hence, T. seems to use the previous experience in his friends’ group to encourage communication in the class and maintain open, porous boundaries:

T: Last year in the class group, during the annual workshop, we had- there was a strong discussion, in which we practically divided in two factions arguing against each other, and we were in the midst of the workshop so we couldn’t even manage it well between us (…) it was a really strong fight. I remember that I had been, I became a bit of a promoter of talking about it, the one who says “ok let’s talk about it though” (…) the reason was a new entry into our class group. We talked about this person and at a certain point I remember I was the one who said, “oh well I’ll throw the bomb, we argued because of this, and therefore it is right that now we talk about it because otherwise we won’t solve anything.” I associate it to what I have said so far about the group of friends, I associate that a little with this.

For his future work as a group therapist, T. also considers this experience useful: “thinking about that experience there, I feel a bit to associate it with some practices that one day I will find myself doing with a therapy group maybe. For example, at that moment, having felt discomfort, not knowing how to manage it, let’s put it this way, I think that eventually it was still useful”.

The discomfort experienced is presented as a useful emotion, as something that has promoted a training need, activated a curiosity, and/or set something in motion. This personal involvement is also recognized by T. as part of the professional competence required to a therapist.

The issue of communicating in a group is taken up again by T. when he talks about the AEC group (cultural educational assistant) chosen for the work area. In this case he emphasizes the importance of *not* communicating, or rather of knowing how to be silent about some things, considering the hierarchy, roles, and institutional dynamics. The following are the aspects that participation in this group highlights:

T.: This is the first time that I worked in a group where we were all under a manager, a director and so on. So, it actually taught me to understand the hierarchy, let’s say, of the work, and to be careful even to the words, the information that is conveyed within the group. It could then maybe get to the wrong ears.

In the interview, T. illustrates an interestingly focused reflection on group participation centered on the issues of conflict management, communication in groups, closed or permeable group boundaries. These topics are brought into play in the different group experiences, each time an aspect is added to the picture and the topic gets more faceted. The quarrel and the inexplicable departure of two members from the first group of friends arises as a critical event at the origin of the trajectory. The discomfort arises as the affect associated with the event.

In displaying a narrative organized in this way, T. present himself as someone capable of self-observation, able to connect events (e.g., the quarrel) with the configuration of the group, able to take into account the emotions experienced as an informative fact, able to share and open up in a joint reflection process. So doing, T. displays himself as competent in the approach promoted by the school and, through this very narrative organization, as a kind of professional who constructs himself through self-reflection. Reflexive narratives are indeed occasioned by the interview. Nevertheless, they are also the culturally situated way of the community of giving shape to one’s initial professional identity.

### D. and the Memorable Rejection

A second example of a learning path across groups is provided by D., 34 years old.

Interviewee D. tells an episode related to his family that he had chosen as a particularly significant group in the private life area. The episode refers to his rejection at the high school final exam. He presents it as a positive episode due to the understanding shown by family members but also describes it as “a bad period”:

D: I failed the final exam in high school, it was a bad period, my mother had just got divorced, in short, a great chaos; this rejection came to me a little unexpectedly in reality because it was a period in which I did not actually notice how I was doing or how I was in school, (…) I remember that the first time I saw my mother, that I saw my aunts, I expected disapproval and I expected a lot of it, instead what came to me was, not consolation, but understanding on their part, for what was happening, perhaps more understanding than what I had at that moment, it was a turning point for me, for my attitude toward studying, it changed me a lot. It was a Sunday, if I remember correctly, it was a Sunday at lunch. I had to repeat the school year but there I pulled myself together a little bit, about what I was doing, where I was going and why, I changed my attitude a lot.

The exam rejection is presented as a consequence of a disruption in the family order (divorce, chaos), and as a failure or a fault that should generate disapproval. Instead, the positive reaction of family members leads to a different reading and to a “turning point”. Subsequently in the interview, D. refers to this experience in the family group to explain his behavior in the group of the graduate school class; influenced by the “memorable rejection”, he later adopted a very studious attitude, which sometimes isolated him in the class:

D: The first year was a bit complex (…) there were cohesion problems in the group. It was quite a difficult moment, emotionally, for the whole group or for almost everyone in the whole group. To be true I was less in this type of emotion, I was more, I was in a phase like “okay we study we work we graduate,” but, mindful of the memorable rejection, “above all we study!” (laughs) this thing quite bothered the rest of the group; they were more on an emotional stance.

The narrative of D. concerns demanding issues such as failure, judgment, and guilt. It originates in the family group and in the critical event of failing the high school exam, which is presented as both a positive and a negative event at the same time: difficult and painful but also evolutionary and promoter of change. Following this event, D. changes his attitude toward studying, perhaps pushing himself too much in the opposite direction in a way that was unwelcomed by the school class (“that kind of attitude that afterward I realized was not the most functional in that moment”). The narrative of D. well shows how in this learning process there is no point of arrival, but rather continuous adjustments to contexts. His narrative also points at group dynamics (in the family, the class) which are used to interpret events and his own behavior. By adopting such a two-fold focus on both contextual dynamics and himself, D. discursively constructs his identity as the outcome of a negotiating and intersubjective process.

### V. and the Risks of Leadership

Another example of reflexive narrative on learning practices across groups is provided by V., 31 years old. Explicitly, V. links the experience as the first born in the family group and that of being project leader in a therapeutic community:

V:(…) The relationship with my brothers, in which I was a bit the leader of the group, of the subgroup (…) let’s say this leadership brought with it issues of responsibility, positive issues and negative issues, that I then carried as baggage in the rest of my life. For example, it occurs to me that at work I am easily offered tasks of responsibility. In facing these proposals, I have sometimes pulled back because I have learned in the family group how being responsible, having responsibilities, being a leader brings complex issues with it, it is not only pleasantness but also responsibility and risk.
R: Risk in what sense?
V: Well because when you have a job responsibility- let’s, for example, think of the community where I work. The role of project manager represents an important role with respect to a relationship with patients, with respect to thinking about the project to be built, but it is also a risk because then you have the responsibility to put your personal signature on it and then if you make a mistake, in that case, it can mean a lot, it means taking responsibility for what happens for the boy, and I learned this at home, especially in moments in which I felt I was put in the middle by my parents, within the problems between my parents and my brothers. So, in this sense responsibility is positive but also negative and risky.

Subsequently in the interview, he tells of a work assignment in which he had to manage a therapeutic group in particularly difficult conditions. He felt unprepared and refers of the burden of the anxiety: “that session was very heavy for me, it was all on me!” This was followed by an interruption of the experience and a renegotiation of the work conditions with the therapeutic community.

The narrative of V. deals with the issue of responsibility in a group, highlighting all the above-mentioned emotional weight, the risks of error, and his safeguarding strategy of backing out. The critical events at the origin of the trajectory are the family quarrels in which he was put in the middle between the parents and the younger siblings. Interviewee V. connects current work assignments and the ways he manages and feels them to his participation in the family group. In doing this, V. discursively presents himself as the result of these multiple and demanding participations.

## Conclusion

This article contributes to describe and to analyze the learning processes as situated and jointly constructed through group participation over time. Data from interviews show how the experience made in groups of different practices is elaborated in and through narratives, highlighting it as a fundamentally collective and culturally shaped sense-making process. Occasioned by the interview, reflexive and meaningful self-narratives stand out as the culturally situated way of the community in giving shape to the formation of professional identity as the outcome of multiple and demanding group participations. Our study described the narrative forms through which professional identity is shaped. The psychoanalytic trainees discursively build themselves as reflexive and self-aware professionals who are competent in reading the implications of their group participations. They do it by elaborating in certain ways on what they have learned and by connecting group experiences in the different realms of private, educational, and work life. The organization of their narratives displays a focus on processes and practices that goes beyond the formal or the temporal group boundaries and a stance that values the comparison and connection of different experiences.

For the psychoanalytic trainees, self-awareness within relationships is an important expert practice to develop, which is, in fact, promoted during training in several ways, including through a personal psychoanalytic therapy.

Moreover, our study contributes to a better understanding of learning processes which happen across groups. We empirically show that, by participating in a group, people not only learn the practices of that group but also develop a sort of meta-learning which takes place across groups. In particular, the interviewees faced similar problems in different groups and use similar practices to manage them. In moving from one group to another, however, there is an evolution, a learning, and a modification “*in situ*” of these practices, which became part of a personal repertoire to be used in other and different groups. This study contributes to the literature on situated learning by showing some features of the personal learning paths of the psychoanalytic trainees across groups. In particular, their narratives appear to be organized around complex topics of group participation; they begin in early group experiences, are associated with a critical event and a negative effect, are in continuous evolution and construction, proceeding by subsequent adjustments in relation to the different groups. Our study points to the emotional strain that the interviewees endure in these early group experiences to manage the complexities of group life. At the same time, it seems that these critical events are exactly what trigger and sustain learning trajectories. Future research might look further into these “crises”, investigating in more detail how such learning paths originate and how affect, motivation and cognition are involved in these moments.

Our data also show how, through experiences of participation in groups over the course of life, future therapists progressively elaborate ways and practices of managing the complexities of being in groups. However, not all groups contributed equally to promoting the learning of professional practices. In particular, the groups with the highest formative potential were found to be those: (1) transversal to several areas (they combine private life, education, and work); (2) which have a certain duration and continuity over time; this allows for the creation of a sense of belonging, a history of participation, and formation of bonds between the participants; (3) which involve informal shared moments and events. The graduate school class, which is also transversal to the educational groups set up by the school, revealed a strong educational potential, depicting itself as something that is much more than a simple grouping of students who followed the courses together for four years.

These analyses might lead to rethinking the characteristics of some learning contexts for group psychotherapists. A first hint is to overcome the distinction between formal educational contexts and other life contexts, and to consider, instead, the outlined properties of the groups: transversality, duration/attendance, and informality. In our data, groups with these characteristics seem to produce a high level of engagement. Further research and reflection are needed to explore how to create educational configurations – in and out of the school- that could exploit these group characteristics for the benefit of the training of the professionals. Moreover, further studies should verify whether these formative characteristics are found in the group experiences of other professionals, besides group psychotherapists.

Finally, it is interesting to notice how the interview, besides being a tool for data collection, turned out to be also an opportunity for self-reflection, as some of the interviewees pointed out. Similar kinds of encounter can potentially play a relevant role in the training path of the psychoanalytic trainees, by fostering the capacity to build up and by collectively reflecting upon the building up of meaningful self-narratives on group participation and on identity construction as the result of learning in different communities of practice.

## Data Availability Statement

The original contributions presented in the study are included in the article/supplementary material, further inquiries can be directed to the corresponding author.

## Ethics Statement

The studies involving human participants were reviewed and approved by Ethical committee of the Department of Social and Developmental Psychology, Sapienza University of Rome. The patients/participants provided their written informed consent to participate in this study.

## Author Contributions

FA provided the conception and design of the study, the data collection and analysis, the drafting of the article, and revised it critically for final submission. CZ contributed to the design of the study, the data collection, and analysis. MF revised the article critically for important intellectual content. All authors contributed to the article and approved the submitted version.

## Conflict of Interest

The authors declare that the research was conducted in the absence of any commercial or financial relationships that could be construed as a potential conflict of interest.

## Publisher’s Note

All claims expressed in this article are solely those of the authors and do not necessarily represent those of their affiliated organizations, or those of the publisher, the editors and the reviewers. Any product that may be evaluated in this article, or claim that may be made by its manufacturer, is not guaranteed or endorsed by the publisher.

## Footnotes

1 At the time of the interview, two students recently discussed the final dissertation and formally ended the program.

## Appendix

**Interview guide**

- Socio-demographic information (age, gender, work activity, previous education).

1. The research aims to explore how the experiences of participation in groups affect the construction of the professional competence of a psychotherapist. With this in mind, please list the most significant groups that populate your life or have populated it in recent years, considering both working and educational areas as well as private life and free time.

2. Please, now choose three groups, one for each area (working life, education, free time / private life). Then I will ask you for some information on each of these groups.

(To be repeated for each of the three groups):

3. What is the aim of the group?

4. Who and how many are the members?

5. Which are the main group activities?

6. When/how did you join the group? When/how did you leave? How long di your participation last?

7. How do you recognize a member of the group from one who is not?

8. Sometimes groups speak some sort of familiar lexicon; do you have typical terms or idioms that others outside the group don’t use? Please provide some examples.

9. What did you learn by participating in this group?

10. Tell a positive and a negative episode related to the experience of participating in the group.

11. If you had to give advice to a friend of yours who will join that group, what would you tell him?

12. Are there practices (i.e., ways of doing, ways of reasoning, tools, reflections) that you have learned in this context and also used elsewhere? If so, which ones and where?

13. How useful do you find participating in this group for your training as a psychotherapist? On a scale of 1 to 4 where 1 = nothing, 2 = little, 3 = somewhat, 4 = a lot.
